# Total Penectomy for Recurrent Chordoma of the Corpus Cavernosum

**DOI:** 10.1155/2020/5498069

**Published:** 2020-02-13

**Authors:** Lennert Eismann, Sabine Kess, Christian G. Stief, Frank Strittmatter

**Affiliations:** ^1^Department of Urology, Ludwig-Maximilians-University, Munich, Germany; ^2^Department of Gynecology and Obstetrics, University Hospital of Heidelberg, Heidelberg, Germany

## Abstract

Chordomas are rare low malignant neoplasm arising from remnants of the notochord with predilection site of the clivus or the os sacrum. Standard therapy is radical excision and adjuvant radiation. Due to invasive growth and adjacent to vital structures resection is often incomplete, and therefore, local recurrence is frequent. First, to the best knowledge of our authors, we present a 70-year-old man with a recurrent chordoma infiltrating the corpus cavernosum. Asymptomatic recurrence was diagnosed by magnet resonance imaging according to the standard follow-up. Our interdisciplinary tumor board recommended surgical resection. We performed a total penectomy and perineal urethrostomy to achieve negative resection margins and preserve best quality of life for the patient.

## 1. Introduction

Chordoma is a rare malignancy with an incidence reported up to 0.08/100,000 person per year with a predominance in male patients according to the Surveillance, Epidemiology, and End Results (SEER) [1]. They arise from the notochord along the axial skeleton and occur most frequently in the os sacrum and the skull base region [1]. According to the histopathology, chordomas are considered low-grade malignant neoplasms; therefore, slow but locally aggressive growth patterns explain late clinical appearance [2]. Independent of primary tumor location, standard therapy remains radical en bloc resection [3]. Total resection is often not feasible due to infiltration of surrounding vital structures [1]. Local recurrence is frequently seen after incomplete resection or after violation of tumor capsule [1, 4]. The 5-year local relapse rate for completely resected sacral chordoma remains 30% [5]. Local recurrence determines patient's survival [1]. In 5% of patients suffering from chordomas, distant metastasis in the lungs, bone, skin, and the central nervous system are reported at initial diagnosis [1].

Here, we report about a rare case of local recurrence of a sacral chordoma after multiple resections. The patient was referred to our urological department for surgical treatment of a clinically inapparent recurrence located on the basis of the corpora cavernosa. We performed a complete resection of the recurrent chordoma by total penectomy and perineal urethrostomy.

## 2. Case Presentation

A 70-year-old patient was referred to our Department of Urology, Faculty of Medicine, Munich Germany, for a surgical treatment of a recurrent chordoma located at the radix penis. The primary chordoma of the os sacrum was diagnosed in 1996 and primarily resected. In the following eight years, the patient underwent three resections (R2) of local recurrences before starting medical therapy with imatinib 400 mg per day in 2009. In 2014, a dorsal complete compartment resection was performed for a fifth local recurrence (R0). Imatinib was paused until in the same year, a single pulmonary metastasis was diagnosed and treated by surgery (R0). Consequently, the medical therapy with imatinib was restarted. In the following year, a local recurrence of the proximal femur was diagnosed. Furthermore, the complete resection (R0) of the gluteal metastases was performed in 2017. In February 2019, another pulmonary metastasis was treated by atypical pulmonary resection (R0).

**Figure 1:**
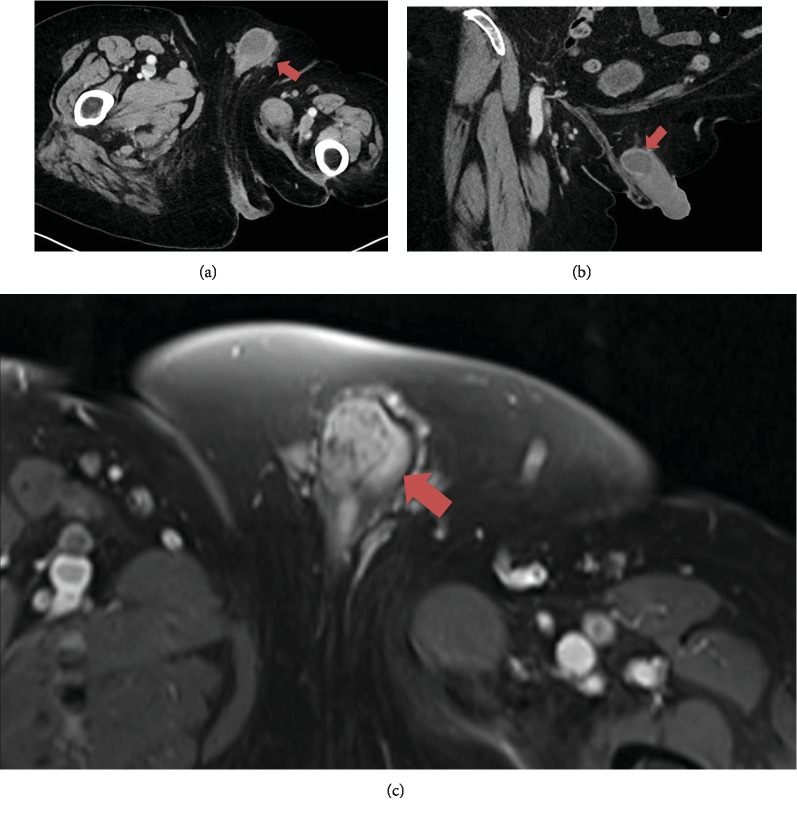
Preoperative MR imaging: (a) axial, (b) coronal, and (c) axial in T1 perfusion. Red arrows mark chordoma metastasis at the right corpus cavernosum of the penis as solid mass of about 2.3 × 3.0 cm.

The follow-up computer tomography (CT) and magnet resonance imaging (MRI) indicated a suspicious lesion of the radix penis (Figure 1). Therefore, a biopsy was taken to prove the seventh local recurrence. The histopathology showed a chondroid chordoma. Clinically, the recurrence was unapparent. The interdisciplinary tumor board advised a surgical treatment.

### 2.1. Surgical Treatment

**Figure 2:**
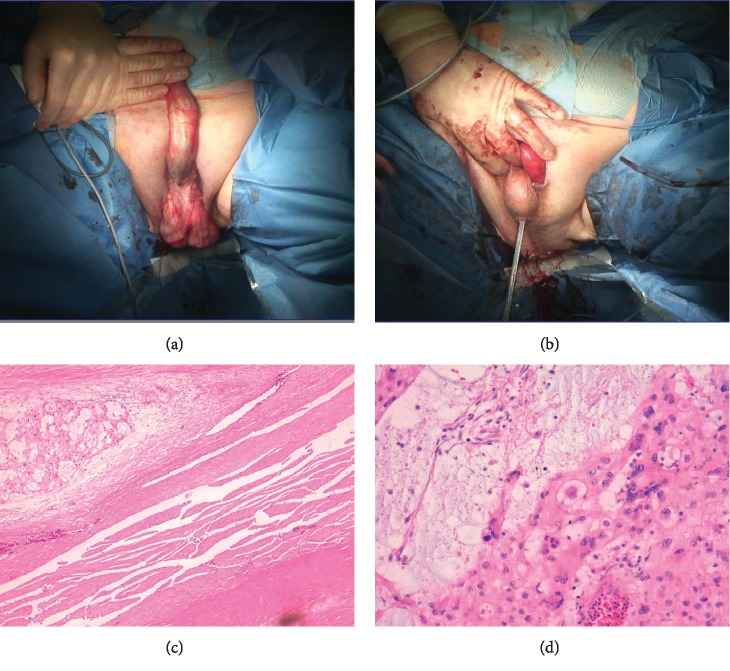
(a) First, the penis was denudated and the corpora cavernosa was separated before complete penectomy was performed. (b) In the last step, the urethra was diverted through the perineum. In the histology findings, the hematoxylin and eosin section shows corpora cavernosa infiltrated by typical physaliferous vacuolated large chordoma cells embedded in a myxoid stroma in images (c) and (d) (magnification 20x).

For preoperative preparation, a standard blood sample was taken, an abdominal CT scan in order to plan surgical procedure was performed and, antibiotic perioperative prophylaxis was given. We performed a total penectomy and perineal urethrostomy (Figures 2(a) and 2(b)). The intraoperative surgical margin was negative. Postoperatively, a transurethral catheter was used for 5 days to decrease risk of infection and to improve wound healing. A total inpatient stay of 7 days was noted. We reevaluated wound healing and functional outcome 14 days postoperatively.

### 2.2. Histopathology Findings

The macroscopic preparation showed an asymmetric indurated lesion with a maximal expansion of 4.1 × 2.5 × 2.6 cm. The resection margin to the corpus cavernosum was measured 0.3 cm and to the corpus spongiosum 1.9 cm. The tumor showed a suppressive, but not infiltrative growth of the corpus cavernosum.

The microscopic evaluation revealed a microcystic and partly solid tumor embedded in a myxoid and chondroid matrix (Figures 2(c) and 2(d)). In the center of the tumor, hemorrhagic and necrotic parts were identified. The tumor cells were voluminous and rich of vacuoles with rough chromatin.

## 3. Discussion

Local recurrence of chordoma is common even after aggressive surgical excision of primary tumor depending on surgical margins [4, 6]. Incomplete resection in initial therapy is a prognostic predictor for poor overall survival [7]. The literature describes 49% of disease-free survival after 15 years for negative surgical margins in comparison to 7% after R1 status in sacral chordomas [5]. Postoperative follow-up to detect recurrence is important before reaching a size to become clinically apparent [8]. The treatment of recurrence remains the surgical resection and/or radiotherapy [4]. The literature describes that local recurrence of the os sacrum and mobile spine is highly related to tumor-related death [9]. Treatment options have to be chosen carefully by an interdisciplinary team to preserve best quality of life [4].

Most penile tumors are squamous cell carcinoma [10]. Metastases of the penis are uncommon, most frequently are primary malignancies from the urogenital tract [11]. Depending on tumor stage and location of the tumor, there are various treatment options [10]. The gold standard in advanced tumor stage is total penectomy and perineal urethrostomy [12]. Also, perineal urethrostomy is the last treatment option for benign urethral disease such as urethral strictures [13]. Despite invasiveness of total penectomy particularly affecting sexuality [14], Barbagli et al. reports a satisfaction after perineal urethrostomy in cases of urethral stricture of 78% [15].

There is one case that reported of a primary vaginal chordoma treated successfully by surgery; other than that, no chordomas of the genital region have been described so far [16].

We describe the first case of a recurrent chordoma located at the corpus cavernosum treated with total penectomy and perineal urethrostomy. For best oncological outcome, wide resection margins were achieved and perineal urethrostomy was performed to preserve continence.

## 4. Conclusions

The treatment of chordoma and its recurrence disease demands an aggressive surgical excision for best long-term survival in consideration of conserving vital structures and preserving best quality of life.
